# Supplementary material for: Evaluation of Candidate Reference Genes for Gene Expression Analysis in Wild Lamiophlomis rotata
Source: Genes (Basel). 2023 Feb 24;14(3):573. doi: 10.3390/genes14030573 (PMC10048348; doi:10.3390/genes14030573)
Supplement: Supplementary file 1 [file genes-14-00573-s001.zip › genes-2200285-supplementary.pdf]

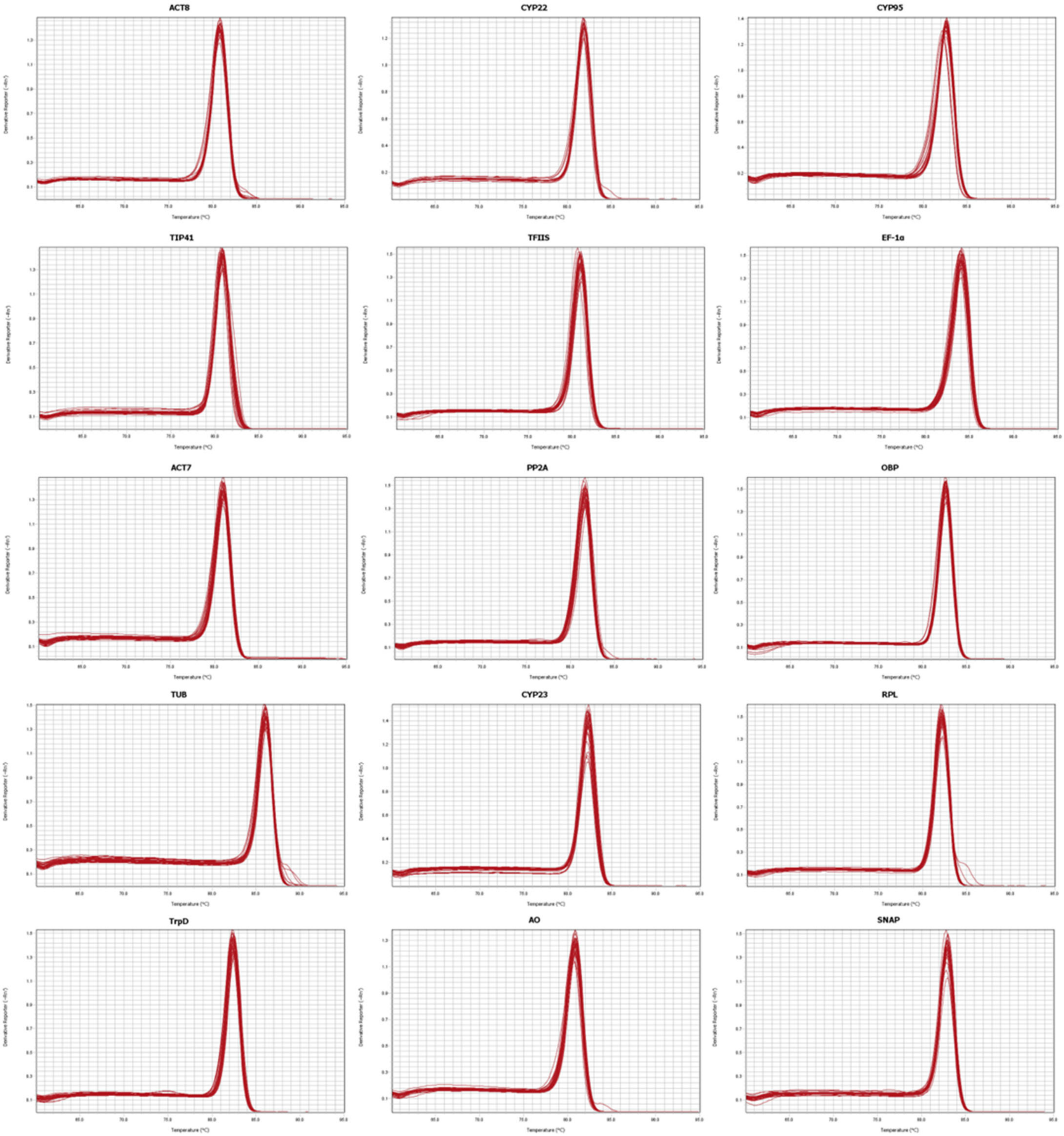

Figure S1. Melting peaks of candidate reference genes. The melting curve of every reference gene by RT-qPCR is a single peak, which indicates the specificity and reliability of the primer pair used for the further analysis of gene expression.
